# New phylogenomic data support the monophyly of Lophophorata and an Ectoproct-Phoronid clade and indicate that Polyzoa and Kryptrochozoa are caused by systematic bias

**DOI:** 10.1186/1471-2148-13-253

**Published:** 2013-11-17

**Authors:** Maximilian P Nesnidal, Martin Helmkampf, Achim Meyer, Alexander Witek, Iris Bruchhaus, Ingo Ebersberger, Thomas Hankeln, Bernhard Lieb, Torsten H Struck, Bernhard Hausdorf

**Affiliations:** 1 Zoological Museum, University of Hamburg, Martin-Luther-King-Platz 3, D-20146 Hamburg, Germany; 2 School of Life Sciences, Arizona State University, 427 East Tyler Mall, Tempe, AZ 85287, USA; 3 Institute of Zoology, Johannes Gutenberg University, J-J Becher-Weg 7, D-55128 Mainz, Germany; 4 Institute of Molecular Genetics, Biosafety Research and Consulting, Johannes Gutenberg University, J-J Becherweg 32, D-55099 Mainz, Germany; 5 Bernhard Nocht Institute for Tropical Medicine, Bernhard-Nocht-Str 74, D-20359 Hamburg, Germany; 6 Department for Applied Bioinformatics, Institute for Cell Biology and Neuroscience, Goethe University, Max-von-Laue-Str 13, D-60438 Frankfurt, Germany; 7 Zoologisches Forschungsmuseum Alexander Koenig, Adenauerallee 160, D-53113 Bonn, Germany

**Keywords:** Bryozoa, Brachiopoda, Brachiozoa, Ectoprocta, Lophophorata, Phoronida, Polyzoa, Kryptrochozoa, Compositional bias

## Abstract

**Background:**

Within the complex metazoan phylogeny, the relationships of the three lophophorate lineages, ectoprocts, brachiopods and phoronids, are particularly elusive. To shed further light on this issue, we present phylogenomic analyses of 196 genes from 58 bilaterian taxa, paying particular attention to the influence of compositional heterogeneity.

**Results:**

The phylogenetic analyses strongly support the monophyly of Lophophorata and a sister-group relationship between Ectoprocta and Phoronida. Our results contrast previous findings based on rDNA sequences and phylogenomic datasets which supported monophyletic Polyzoa (= Bryozoa sensu lato) including Ectoprocta, Entoprocta and Cycliophora, Brachiozoa including Brachiopoda and Phoronida as well as Kryptrochozoa including Brachiopoda, Phoronida and Nemertea, thus rendering Lophophorata polyphyletic. Our attempts to identify the causes for the conflicting results revealed that Polyzoa, Brachiozoa and Kryptrochozoa are supported by character subsets with deviating amino acid compositions, whereas there is no indication for compositional heterogeneity in the character subsets supporting the monophyly of Lophophorata.

**Conclusion:**

Our results indicate that the support for Polyzoa, Brachiozoa and Kryptrochozoa gathered so far is likely an artifact caused by compositional bias. The monophyly of Lophophorata implies that the horseshoe-shaped mesosomal lophophore, the tentacular feeding apparatus of ectoprocts, phoronids and brachiopods is, indeed, a synapomorphy of the lophophorate lineages. The same may apply to radial cleavage. However, among phoronids also spiral cleavage is known. This suggests that the cleavage pattern is highly plastic and has changed several times within lophophorates. The sister group relationship of ectoprocts and phoronids is in accordance with the interpretation of the eversion of a ventral invagination at the beginning of metamorphosis as a common derived feature of these taxa.

## Background

The evolution of metazoan body plans remains highly controversial due to persisting uncertainty regarding the phylogeny of major animal clades. In this context, the phylogenetic position of the three lophophorate lineages, namely ectoprocts, brachiopods and phoronids, which are mainly marine invertebrates characterized by an eponymous filter apparatus, has proven to be particularly elusive. Based on ontological and morphological data, they were initially considered the sister or, alternatively, the paraphyletic stem-group of Deuterostomia [1-5]. However, molecular phylogenetic analyses changed our views about the relationships of the lophophorate lineages. Based on analyses of 18S rDNA sequences, Halanych et al. [6] were the first to recognize that the lophophorate lineages are more closely related to Annelida and Mollusca than to deuterostomes. As a consequence, they united Lophophorata and Trochozoa to form Lophotrochozoa. Since then, the monophyly of Lophotrochozoa has been confirmed by further analyses of rDNA sequences [7-13], single nuclear protein-encoding genes (e.g., [14,15]), *Hox* genes [16,17], mitochondrial protein sequences [18-24], multiple nuclear protein-encoding sequences [25,26] and by phylogenomic approaches [27-35]. The only potential morphological apomorphy of Lophotrochozoa found so far is a larval apical organ with serotonin expressing flask-shape cells [36,37].

While the monophyly of the Lophotrochozoa has meanwhile been widely accepted, the discussion concerning the phylogenetic relationships within Lophotrochozoa is still ongoing. Halanych et al. [6] suggested that lophophorates are polyphyletic, because ectoprocts formed the sister group of all other lophotrochozoans in their tree. Moreover, they proposed that phoronids are the sister clade of articulate brachiopods, making brachiopods also paraphyletic. It turned out that their clustering of phoronids and articulate brachiopods was an artifact probably caused by a chimeric sequence [38]. Still, the monophyly of Brachiozoa (=Phoronozoa) including brachiopods and phoronids was later independently corroborated by analyses based on rDNA [7,8,12,13,38-41] and sodium-potassium ATPase α-subunit sequences [15], multiple nuclear protein-encoding sequences [26,42], total evidence analyses [9,39,43] and phylogenomic approaches [30,35]. The relationships within Brachiozoa are, however, in dispute. Whereas some rDNA analyses indicate that brachiopods are paraphyletic and phoronids are the sister group of inarticulate brachiopods [38,40,41,44], brachiopods come out as monophyletic in analyses of morphological data [2,4,9,39,43,45-47], of multiple nuclear protein-encoding sequences [42], and of phylogenomic datasets [30,35].

Furthermore, phylogenomic analyses suggested that phoronids and brachiopods form a clade with nemerteans [28-30,35,48,49], named Kryptrochozoa [48]. Finally, phylogenomic analyses indicated that ectoprocts are the sister group of entoprocts and cycliophorans. As a consequence, the old Polyzoa (=Bryozoa sensu lato) hypothesis was revived [27,28,30-34], which has been supported by a few morphologists [47], and which has recently also been corroborated by analyses of rDNA sequences [12,13], albeit with weak support.

The relationships of Kryptrochozoa and Polyzoa to other lophotrochozoan phyla could, so far, not be decisively resolved. This is despite the fact that numerous EST and genome projects have resulted in an improved taxon sampling and an increase of the number of available genes [27-34]. While phylogenomic studies are likely to reduce the influence of random errors and gene specific influences on phylogenetic inference [50], they cannot cope with the fact that model violations such as compositional biases in the data can confound accurate tree reconstruction [51-53]. That a biased amino acid composition indeed affects phylogenetic analyses of the metazoan phyla has been demonstrated by Nesnidal et al. [35]. Two main strategies have been proposed for dealing with compositional heterogeneity in the data. The most straightforward procedure is the exclusion of particularly affected partitions from the analysis. Alternatively, one can rely on phylogeny reconstruction methods that can account for compositional heterogeneity, and thus ameliorate their confounding influence. The outcome of the tree reconstruction varies with the chosen method to cope with the bias. Whereas some approaches supported the monophyly of Polyzoa including ectoprocts and entoprocts, other strategies, such as the exclusion of taxa with the most deviating amino acid composition surprisingly revealed monophyletic Lophophorata [35].

In this study we investigated the relationships among the lophophorate lineages and other lophotrochozoans together with potential sources of systematic errors that might affect these phylogenetic analyses, namely contaminations, incorrect orthology assignments and compositional bias. We base our analyses on a new dataset comprising 196 proteins from 58 bilaterian taxa.

## Results and discussion

### Relationships of the lophophorate lineages

**Figure 1:**
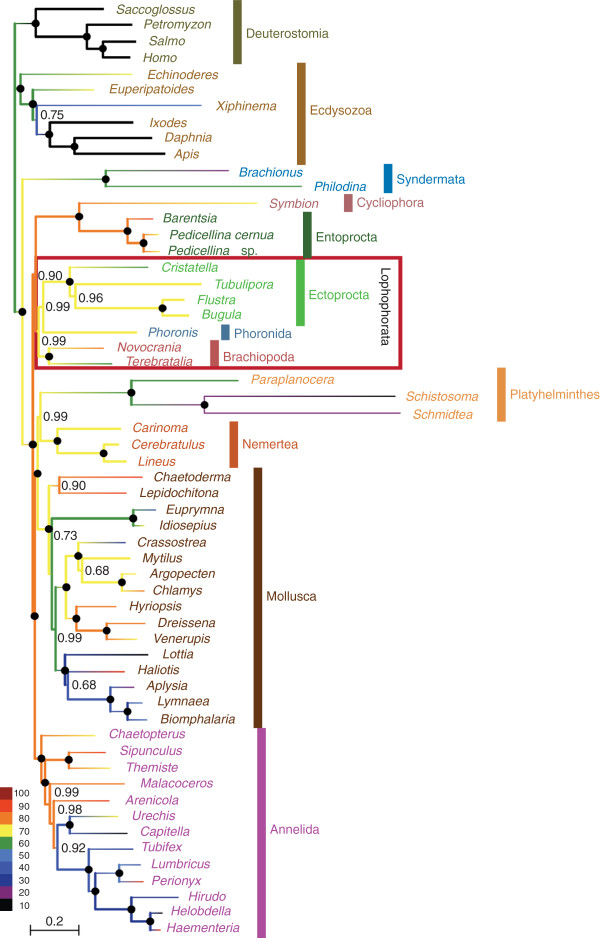
**Bayesian inference reconstruction with the CAT model based on 41,292 amino acid positions derived from 196 proteins of 58 taxa.** Bayesian posterior probabilities are shown to the right of the nodes; posterior probabilities equal to 1.0 are indicated by black circles. The colour of the branches visualizes the percentage of missing data.

**Figure 2:**
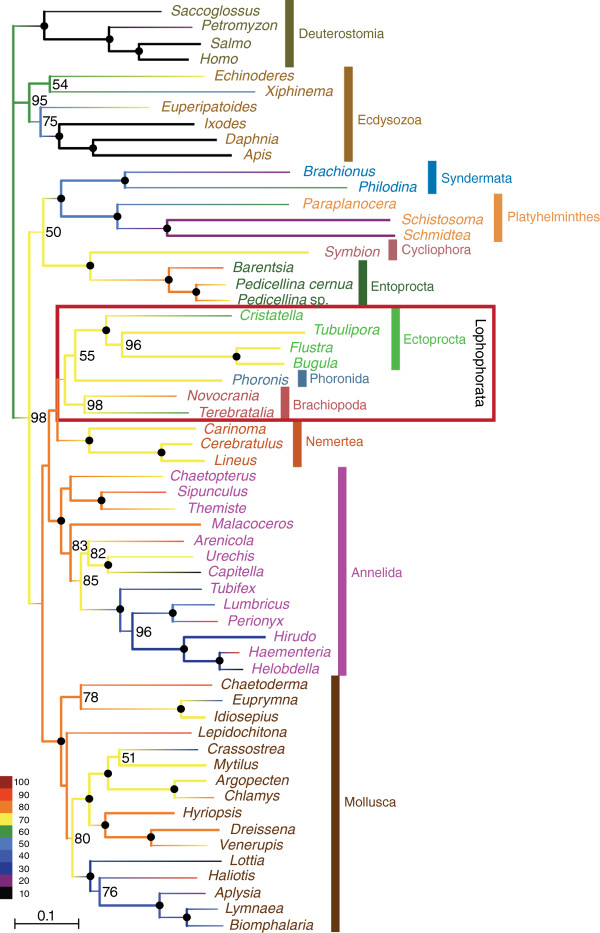
**Maximum likelihood tree calculated with the LG+G+F model based on 41,292 amino acid positions derived from 196 proteins of 58 taxa.** Bootstrap values larger than 50% are shown to the right of the nodes; 100% bootstrap values are indicated by black circles. The colour of the branches visualizes the percentage of missing data.

**Figure 3:**
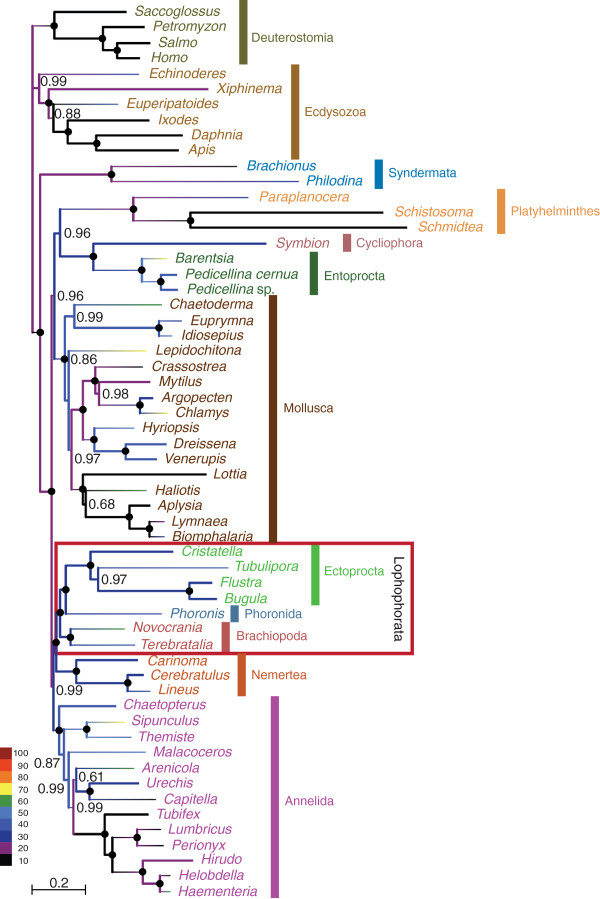
**Bayesian inference reconstruction with the CAT model based on 15,849 amino acid positions of 58 taxa.** Bayesian posterior probabilities are shown to the right of the nodes; posterior probabilities equal to 1.0 are indicated by black circles. The colour of the branches visualizes the percentage of missing data.

**Figure 4:**
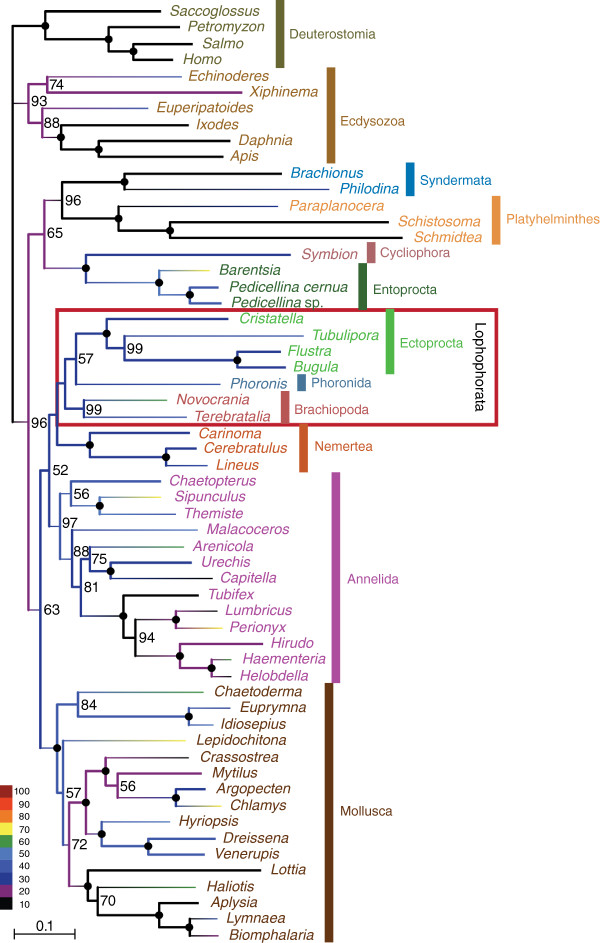
**Maximum likelihood tree calculated with the LG+G+F model based on 15,849 amino acid positions of 58 taxa.** Bootstrap values larger than 50% are shown to the right of the nodes; 100% bootstrap values are indicated by black circles. The colour of the branches visualizes the percentage of missing data.

The complete dataset that we compiled for the phylogenomic analysis of the relationships of the lophophorate lineages comprised 196 genes from 58 metazoan taxa. The corresponding super-alignment spans 41,292 amino acid positions and has 50.4% data coverage. A PhyloBayes analysis of this dataset with the CAT model (Figure 1) revealed strong support for the monophyly of Lophophorata (Bayesian posterior probability (BPP): 0.99) and the monophyly of Ectoprocta + Phoronida (BPP: 0.99). A maximum likelihood analysis with the LG model (Figure 2) confirmed these relationships, albeit without statistical support or only weak support (bootstrap support (BS) for Lophophorata: 37%; for Ectoprocta + Phoronida: 55%). A selection of those positions from the complete dataset where data are available from at least 50% of all included taxa increased data coverage to 72.4%. The percentage of known character states increased especially in the less well-covered smaller phyla that are the focus of our study (compare the colour coding of the branches in Figures 1 and 2 versus Figures 3 and 4). This can also been seen in density distributions of shared missing data, which is strongly shifted to lower values in the reduced dataset (compare Additional file 1: Figure S1 and Additional file 2: Figure S2). Phylogenomic analyses of this dataset encompassing 15,849 sites (Figures 3 and 4) confirmed the monophyly of Lophophorata (BPP_red_: 1.00; BS_red_: 37%) and the monophyly of Ectoprocta + Phoronida (BPP_red_: 1.00; BS_red_: 57%) and, thus, show that these groupings are not artifacts resulting from the amount of missing data. However, rather than based solely on the amount of missing data artificial signal for a grouping of taxa might also stem from a strong degree of overlap in missing data shared between taxa, if the missing data are not randomly distributed across the taxa, but are systematically biased [54-56]. Hierarchical clustering analyses based on the degree of overlap in missing data shared between taxa (Additional file 1: Figure S1 and Additional file 2: Figure S2) corroborate that neither Lophophorata nor Ectoprocta + Phoronida are artifacts caused by shared missing data. The taxa belonging to these groups do not cluster in these analyses, but are scattered among other lophotrochozoan taxa.

These results challenge the Brachiozoa, Polyzoa (=Bryozoa sensu lato) as well as Kryptrochozoa hypotheses. Interestingly, a sister group relation between ectoprocts and phoronids had been previously proposed based on morphological data [57,58]. The PhyloBayes analysis with the reduced dataset (Figure 3) and the maximum likelihood analyses support a sister group relationship between Lophophorata and Nemertea (BPP: 1.00; BS: 38%; BS_red_: 46%). In contrast, the PhyloBayes analysis with the complete dataset (Figure 1) indicates that Entoprocta + Cycliophora might be the sister group of Lophophorata (BPP: 0.90) and that Nemertea and Platyhelminthes are sister groups (BPP: 0.99) as has previously been suggested based on morphological data (‘Parenchymia’ hypothesis [4,47], but see [31]).

### Causes of incongruent topologies

The results of our phylogenetic analyses are incongruent with those of previous phylogenomic analyses, which revealed monophyletic Polyzoa [27,30-35], Brachiozoa [30,35] and Kryptrochozoa [28-30,35,48,49]. These incongruences cannot be ascribed to random errors, since the monophyly of Polyzoa, Brachiozoa and Kryptrochozoa was strongly supported in most of the previous phylogenomic analyses, whereas the mutually exclusive monophyly of Lophophorata and Ectoprocta + Phoronida is strongly supported in the PhyloBayes analyses with the CAT model (Figures 1, 3). The hierarchical clustering analyses based on degrees of overlap in missing data shared between taxa (Additional file 1: Figure S1 and Additional file 2: Figure S2) also showed that similar to Lophophorata and Ectoprocta + Phoronida Polyzoa, Brachiozoa and Kryptrochozoa cannot be attributed to shared missing data as the taxa belonging to these groups are scattered throughout the tree and do not cluster. Therefore, we checked whether these incongruences might be caused by contaminations, incorrect orthology assignments or by compositional bias.

**Figure 5:**
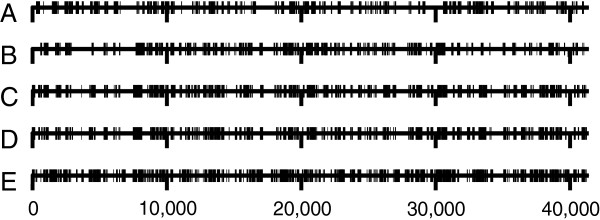
**Distribution of autapomorphies for different taxa across the concatenated alignment. (A)** Lophophorata, **(B)** Ectoprocta + Phoronida, **(C)** Polyzoa, **(D)** Brachiozoa, **(E)** Kryptrochozoa.

We investigated the possibility that contaminations or a few paralogs in the sequence data affect the topology with respect to our focal groups. In this instance we would expect that apomorphies of Lophophorata, as determined by a parsimony mapping of the data on the maximum likelihood tree, cluster in only small parts of the alignment. However, Figure 5A shows convincingly that the apomorphies are distributed evenly along the whole alignment. Thus, the support for Lophophorata is not the result of a few contaminations or incorrect orthology assignments. The same holds for the positions supporting the Ectoprocta + Phoronida clade (Figure 5B) as well as for those that support Polyzoa (Figure 5C), Brachiozoa (Figure 5D) and Kryptrochozoa (Figure 5E) in trees in which these groups are constrained to be monophyletic.

**Figure 6:**
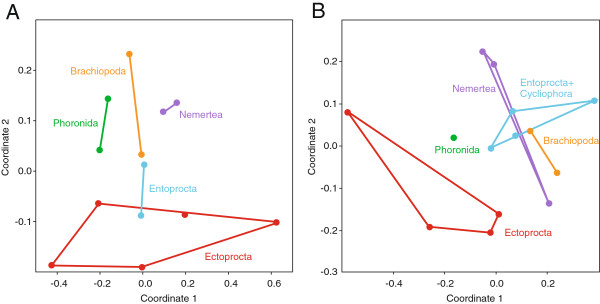
**Non-metric multidimensional scalings of compositional distances between amino acid sequences.** Scaling of distances between focal taxa using **(A)** the ribosomal protein dataset of Nesnidal et al. [35] and **(B)** the dataset used in this study.

As a first step to assess whether a compositional bias might have affected a phylogenomic analysis as asserted by Nesnidal et al. [35], we visualized similarities in the amino acid composition of the focal taxa in non-metric multidimensional scalings. A non-metric multidimensional scaling based on the ribosomal protein dataset of Nesnidal et al. [35] (Figure 6A) shows that the space occupied by the ectoproct sequences overlaps with that occupied by the entoproct sequences, but is clearly separated from that occupied by phoronids, brachiopod and nemertean sequences. In contrast, the space occupied by the ectoproct sequences in the non-metric multidimensional scaling based on the new dataset (Figure 6B) does not overlap with that occupied by entoprocts. These analyses indicate that the Polyzoa clade in the former analyses might have been an artifact resulting from compositional bias.

We investigated this issue further by analyzing the amino acid composition of the character subsets that support the conflicting nodes. The amino acid composition of the reconstructed ancestral sequence of Lophophorata based on the new dataset is not significantly deviating from the overall amino acid composition in the dataset including the 1,005 characters that display apomorphies for Lophophorata (Table 1). The same is true for the ancestral sequence of Lophophorata + Nemertea. Thus, this analysis provides no indication that the monophyly of Lophophorata is caused by a compositional bias. However, the composition of the reconstructed ancestral amino acid sequence of Ectoprocta + Phoronida is significantly deviating from the overall amino acid composition in the dataset including the 1,271 characters that display apomorphies for Ectoprocta + Phoronida (Table 1).

**Table 1:** Composition of the subsets of characters supporting Lophophorata and Ectoprocta + Phoronida in the unconstrained tree and Brachiozoa and Polyzoa in constrained trees

**Hypothesis**		**No. of autapomorphies**	**p-value chi-square test**
Lophophorata		1,005	
	Lophophorata		82.54%
	Lophophorata + Nemertea		89.02%
Ectoprocta + Phoronida		1,271	
	Ectoprocta + Phoronida		0.01%*
	Lophophorata		29.57%
Polyzoa		1,569	
	Polyzoa		0.29%*
	Polyzoa + Brachiozoa		31.24%
Brachiozoa		1,522	
	Brachiozoa		0.00%*
	Lophophorata		8.81%
Kryptrochozoa		1,792	
	Kryptrochozoa		0.00%*
	Kryptrochozoa + Annelida		16.21%

*compositional homogeneity significantly rejected.

To investigate the so far hidden support in our new data for the Polyzoa, Brachiozoa and Kryptrochozoa hypotheses respectively, we constructed maximum likelihood trees enforcing the monophyly of these groups. We then repeated the parsimony mapping and investigated the composition of the apomorphies in supporting these groupings. Polyzoa are supported by 1,569 autapomorphies. The amino acid composition of the reconstructed states of the hypothetical polyzoan ancestor for these positions deviates significantly from the overall composition of all taxa at these positions (Table 1). In contrast, the composition of the reconstructed states of the hypothetical ancestor of Polyzoa and Brachiozoa, the sister group of Polyzoa in the constrained maximum likelihood tree, for this character subset is not significantly deviating (Table 1). This reveals that the amino acid composition of the characters displaying potential autapomorphies for Polyzoa has changed at the base of Polyzoa. In other words, Ectoprocta and Entoprocta + Cycliophora cluster because of character states that differ from those of other taxa in composition and, as a consequence, Polyzoa might be an artifact resulting from compositional bias.

Similarly, Brachiozoa, a clade comprising Brachiopoda and Phoronida, can be attributed to compositional biases. If we constrain the monophyly of Brachiozoa, they are supported by 1,522 autapomorphies. As for Polyzoa, the amino acid composition of the reconstructed ancestral sequence of Brachiozoa is significantly deviating at the positions that display autapomorphies for Brachiozoa (Table 1), whereas the composition of the reconstructed ancestral sequence of Brachiozoa and Ectoprocta, the sister group of Brachiozoa in the constrained maximum likelihood tree, is not significantly different (Table 1).

The same is true for Kryptrochozoa including Brachiopoda, Phoronida and Nemertea. Kryptrochozoa are supported by 1,792 autapomorphies in a tree in which their monophyly is enforced. The amino acid composition of the reconstructed ancestral sequence of Kryptrochozoa is significantly deviating at the positions that display autapomorphies for this clade (Table 1), whereas the composition of the reconstructed ancestral sequence of Kryptrochozoa and Annelida, the sister group of Kryptrochozoa in the constrained maximum likelihood tree, is not significantly different (Table 1).

The fact that the amino acid composition of a character subset changes at a given node does not necessarily mean that it is an artifactual node. However, in the case of the lophophorate lineages, it is more likely that they actually form a monophyletic clade rather than that the conflicting Polyzoa and Kryptrochozoa hypotheses are correct, as we have identified a possible source of systematic error in the data for the latter two hypotheses, but not for the Lophophorata hypothesis. Systematic error resulting from compositional bias might also be the cause of the conflict between the Ectoprocta + Phoronida versus the Brachiozoa hypothesis. However, in this case, both alternatives are potentially affected by compositional bias (Table 1) so that the test for compositional bias does not give a hint which hypothesis corresponds to the true phylogeny.

### Implications for the evolution of morphology

The support for the monophyly of Lophophorata (Figures 1, 2,3,4) indicates that the horseshoe-shaped mesosomal lophophore, the ciliated, tentacular feeding apparatus of ectoprocts, phoronids and brachiopods, is homologous, despite some differences in the structure between these groups [47]. Our results suggest that the epistome, a muscular lobe that is used to push the infiltrated particles into the mouth opening, is a further innovation of the lophophorate lineage [58].

The position of the lophophorates within Lophotrochozoa renders it unlikely that the lophophore of Lophophorata is homologous with the similar tentacular feeding apparatus of the deuterostome Pterobranchia, with which it has been homologized formerly [1,2,47]. Such a homology would require the assumption of multiple, independent transitions from a sessile, filter feeding life style to a mobile life style and associated multiple losses of the tentacular feeding apparatus. However, a sister group relationship between Lophophorata and Entoprocta + Cycliophora as moderately supported by the PhyloBayes analysis with the complete dataset (Figure 1) would imply that a tentacular apparatus for filter feeding as an adaptation to a sessile life style is a synapomorphy of these groups, despite the functional differences between the lophophore of Lophophorata and the tentacular apparatus of Entoprocta [47]. The monophyly of the sessile lophotrochozoan groups with a tentacular feeding apparatus would be much more plausible from a morphological point of view than the Kryptrochozoa hypothesis [28-30,35,48,49] grouping the predatory, vagile nemerteans with the sessile filter feeding brachiopods and phoronids, which have no morphological features in common with nemerteans. However, the conflicting results of our analyses (Figures 1, 2,3,4) indicate that more data are necessary to resolve the interrelationships of Lophophorata, Entoprocta + Cycliophora and Nemertea robustly.

Radial cleavage was formerly considered a symplesiomorphy of lophophorates and deuterostomes [1,2]. However, there are no doubts about the homology of the spiral cleavage of entoprocts, nemerteans, platyhelminths, annelids, and molluscs, the closest relatives of Lophophorata (Figures 1,2,3,4). Taking our phylogeny at face value, parsimony would suggest that radial cleavage evolved secondarily in the lineage leading to the Lophophorata. Alternatively one might assume that Lophophorata is the sister group of the lophotrochozoan phyla that share spiral cleavage. However, the finding that cleavage is spiral in at least some phoronids [59,60] shows how variable cleavage patterns are and that the radial cleavage of lophophorates is probably secondarily derived from spiral cleavage.

Our trees showing a close relationship of Ectoprocta and Phoronida imply that the eversion of a ventral invagination (the metasomal tube in phoronids and the ventral sac in some ectoprocts) at the beginning of the metamorphosis [3,52,61,62] and the loss of setae [61] might be synapomorphies of ectoprocts and phoronids.

Phoronida and Phylactolaemata (Ectoprocta) share a bodywall musculature consisting of a regular grid of an outer layer of circular and an inner layer of longitudinal musculature, whereas Gymnolaemata (=Stenolaemata + Ctenostomata + Cheilostomata), the sister group of Phylactolaemata [2,28,63], and Brachiopoda lack such a distinct regular bodywall musculature [64]. Schwaha and Wanninger [64] discussed whether the similarity of the bodywall musculature of Phoronida and Phylactolaemata evolved convergently or whether Ectoprocta and Phoronida are closely related. Our results support the latter hypothesis. However, a similar bodywall musculature is also found in several other vermiform lophotrochozoan phyla. Thus, it is probably not a synapomorphy for Ectoprocta and Phoronida, but a symplesiomorphy that was lost in Gymnolaemata and Brachiopoda as a result of the evolution of solid exoskeletons.

## Conclusions

Our results support the monophyly of Lophophorata and an ectoproct-phoronid clade and indicate that the support for Kryptotrochozoa and Polyzoa gathered so far is likely an artifact caused by compositional bias. The monophyly of Lophophorata implies that the horseshoe-shaped mesosomal lophophore, the tentacular feeding apparatus of ectoprocts, phoronids and brachiopods is a synapomorphy of the lophophorate lineages. The same may apply to radial cleavage. However, among phoronids also spiral cleavage is known. This suggests that the cleavage pattern is highly plastic and has changed several times within lophophorates. The sister group relationship of ectoprocts and phoronids is in accordance with the interpretation of the eversion of a ventral invagination at the beginning of metamorphosis as a common derived feature of these taxa.

## Methods

### Data sources and orthology assignment

Data were extracted from so far only partly published EST datasets of *Tubulipora* sp. (Ectoprocta), *Flustra foliacea* (Ectoprocta), *Novocrania anomala* (Brachiopoda), *Phoronis muelleri* (Phoronida), *Barentsia elongata* (Entoprocta), *Lineus viridis* (Nemertea) and *Brachionus plicatilis* (Monogononta), of which only the ribosomal protein encoding sequences had yet been used for phylogenetic studies [27,28,30-33]. The EST data used in our analyses have been deposited in the NCBI EST database [65] under accession numbers LIBEST_025704 (*Tubulipora sp.*), LIBEST_028288 (*Flustra foliacea*), LIBEST_028289 (*Novocrania anomala*), LIBEST_028290 (*Phoronis muelleri*), LIBEST_026421 (*Brachionus plicatilis*), LIBEST_027828 (*Barentsia elongata*) and LIBEST_028316 (*Lineus viridis*).

The dataset for tree reconstruction was compiled in a two-step procedure. For the initial ortholog search, we first defined a set of seven species with completely sequenced genomes, the so-called primer taxa: *Caenorhabditis elegans* (Nematoda), *Daphnia pulex* (Crustacea), *Apis mellifera* (Insecta), *Schistosoma mansoni* (Platyhelminthes), *Capitella capitata* (Annelida), *Helobdella robusta* (Annelida) and *Lottia gigantea* (Mollusca). We then used InParanoid-TC [66] to identify genes for which an ortholog was present in each of the seven primer taxa. Finally, we extended the resulting 1,297 ortholog groups (listed in Additional file 1: Figure S1) with sequences from further taxa using HaMStR [66].

### Alignment, alignment masking and gene selection

The amino acid sequences of the 1,297 individual ortholog groups of 58 species were aligned with MAFFT using the most accurate option L-INS-i [67,68]. To increase the signal-to-noise ratio, sections with random sequence similarity were identified with ALISCORE version 1.0 [69,70] and subsequently excluded with ALICUT [71]. We constructed individual trees for each protein using a parallel Pthreads-based version of RAxML version 7.7.1 [72,73] with the LG+G+F model [74] to check for unusual topologies and long branches that might indicate hidden paralogy and contaminations. One gene tree shows a very long, highly supported branch separating a clade including Deuterostomia and Ecdysozoa, into which the three nemertean representatives were nested. This topology is inconsistent with the position of Nemertea within Lophotrochozoa inferred in other analyses and our own analyses, if this protein is excluded. This topology indicates probably a paralogy [75,76]. Thus, we excluded this protein. We also inspected each protein alignment manually for contaminant sequences and poorly conserved motives. Problematic sequences that are difficult to align or result in extraordinarily long branches were excluded from the individual unmasked alignments and all single protein datasets were re-aligned with MAFFT and masked using ALISCORE. All masked alignments that were at least 100 amino acids long and contained at least 25 taxa after the various preprocessing and filtering steps were subsequently concatenated. To assess the effect of missing data, we constructed a reduced alignment by selecting those positions from the basic alignment at which data are available from at least 50% of all included taxa using MEGA version 5.1 [77]. Both superalignments have been deposited at TreeBASE ([78], accession number S13700).

### Phylogenetic analyses

We performed Bayesian inference analyses with the CAT model that adjusts for site-specific amino acid frequencies [79,80] as implemented in PhyloBayes MPI version 1.4f (http://megasun.bch.umontreal.ca/People/lartillot/www/index.htm). For each of the two datsets (complete and reduced) two independent chains were run for 27,500 or 30,000 points, respectively, of which 15,000 or 20,000 were discarded as burn-in. The largest discrepancy observed across all bipartitions (maxdiff) was 0.10 or 0.13, respectively. Taking every 10th sampled tree, a 50%-majority rule consensus tree was computed using both chains of a dataset.

We performed maximum likelihood analyses using a parallel Pthreads-based version of RAxML version 7.7.1 [72,73] with the LG+G+F model [74]. We computed 10 maximum likelihood trees using 10 distinct randomized maximum parsimony starting trees and chose the tree with the highest likelihood. Rapid bootstrapping [81] was used to assess the statistical branch support in the reconstructed phylogeny. We conducted rapid bootstrap analysis and searched for the best-scoring maximum likelihood tree in one single program run. The number of necessary replicates was inferred using the extended majority-rule consensus tree criterion ([82]; 250 replications inferred using the option autoMRE in RAxML).

### Influence of missing data on phylogenetic reconstruction

We visualized the level of missing data in the phylogenetic trees as suggested by Roure et al. [83]. To infer whether the “ancestral” state of a given position for a given node is unknown or known, sequences were recoded with 0’s and 1’s depending on each character state being present or absent. Ancestral sequences were reconstructed by maximum parsimony, using PAUP* version 4.0 beta 10 [84] with the ACCTRAN option, based on the topologies inferred as described above. The percentage of missing data was displayed in the trees by colour coding the branches.

Furthermore, we investigated the influence of shared missing data on phylogenetic reconstruction by hierarchical clustering analyses based on the degree of overlap in missing data between taxa using BaCoCa version 1.105 [85].

### Non-metrical multidimensional scaling of amino acid composition

We visualized similarities in the amino acid composition of the focal taxa in a non-metric multidimensional scaling as implemented in PAST version 2.17c [86] based on compositional distances (one half the sum of squared difference in counts of residues) between taxa calculated with MEGA version 5.1 [77].

### Node based evaluation of potential compositional bias

To investigate whether a node might be affected by compositional bias we determined whether there was a significant shift in the amino acid composition of the apomorphies of this node between the last common ancestor of the clade in question and its direct ancestor. Amino acid substitutions along the tree were traced by parsimony mapping using PAUP* [84]. We retrieved all positions from the dataset, which showed an apomorphy for a specified node. If the node corresponding to the hypothesis to be tested was not present in the unconstrained maximum likelihood tree, we calculated a tree in which the group of interest was constrained to be monophyletic. In addition to the terminal taxa we also included the reconstructed ancestral state of the node in question as well as of the direct ancestor of this node in these subsets. For example, the test for an artificial attraction of Ectoprocta and Phoronida due to a deviating amino acid composition is based on a subset of the alignment comprising the character states at all positions where the ancestor of the Ectoprocta + Phoronida clade is characterized by apomorphies. All terminal taxa and the reconstructed states of the last common ancestor of Ectoprocta and Phoronida as well as its direct ancestor, that is the last common ancestor of all Lophophorata, were considered. Compositional heterogeneity in the alignment subsets was investigated using a chi-square test implemented in TREE-PUZZLE version 5.2 [87].

## Competing interests

The authors declare that they have no competing interests.

## Authors’ contributions

MH, AM, AW, IB, TH, BL and THS generated and provided EST sequences. IE compiled and aligned the ortholog groups. MPN and BH performed the phylogenetic analyses. BH designed the study and drafted the manuscript. All authors contributed to, read and approved the final manuscript.

## Supplementary Material

Additional file 1: Figure S1 Heat map analysis combined with hierarchical clustering of complete dataset of the degree of overlap in missing data shared between taxa. The order of the taxa from left to right along the x-axis is the same as from bottom to top along the y-axis. The higher taxonomic unit of each species is highlighted as indicated in the legend on top. Colours in the heat map indicate proportion of shared missing data ranging from 0 (orange) to 0.8 (red) (see key in upper left corner). The density distribution of the proportions is given in the upper left corner. Click here for file

Additional file 2: Figure S2 Heat map analysis combined with hierarchical clustering of reduced dataset of the degree of overlap in missing data shared between taxa. The order of the taxa from left to right along the x-axis is the same as from bottom to top along the y-axis. The higher taxonomic unit of each species is highlighted as indicated in the legend on top. Colours in the heat map indicate proportion of shared missing data ranging from 0 (orange) to 0.5 (red) (see key in upper left corner). The density distribution of the proportions is given in the upper left corner. Click here for file
