# Supplementary material for: New phylogenomic data support the monophyly of Lophophorata and an Ectoproct-Phoronid clade and indicate that Polyzoa and Kryptrochozoa are caused by systematic bias
Source: BMC Evol Biol. 2013 Nov 17;13:253. doi: 10.1186/1471-2148-13-253 (PMC4225663; doi:10.1186/1471-2148-13-253)
Supplement: Additional file 1: Figure S1 — Heat map analysis combined with hierarchical clustering of complete dataset of the degree of overlap in missing data shared between taxa. The order of the taxa from left to right along the x-axis is the same as from bottom to top along the y-axis. The higher taxonomic unit of each species is highlighted as indicated in the legend on top. Colours in the heat map indicate proportion of shared missing data ranging from 0 (orange) to 0.8 (red) (see key in upper left corner). The density distribution of the proportions is given in the upper left corner. [file 1471-2148-13-253-S1.pdf]

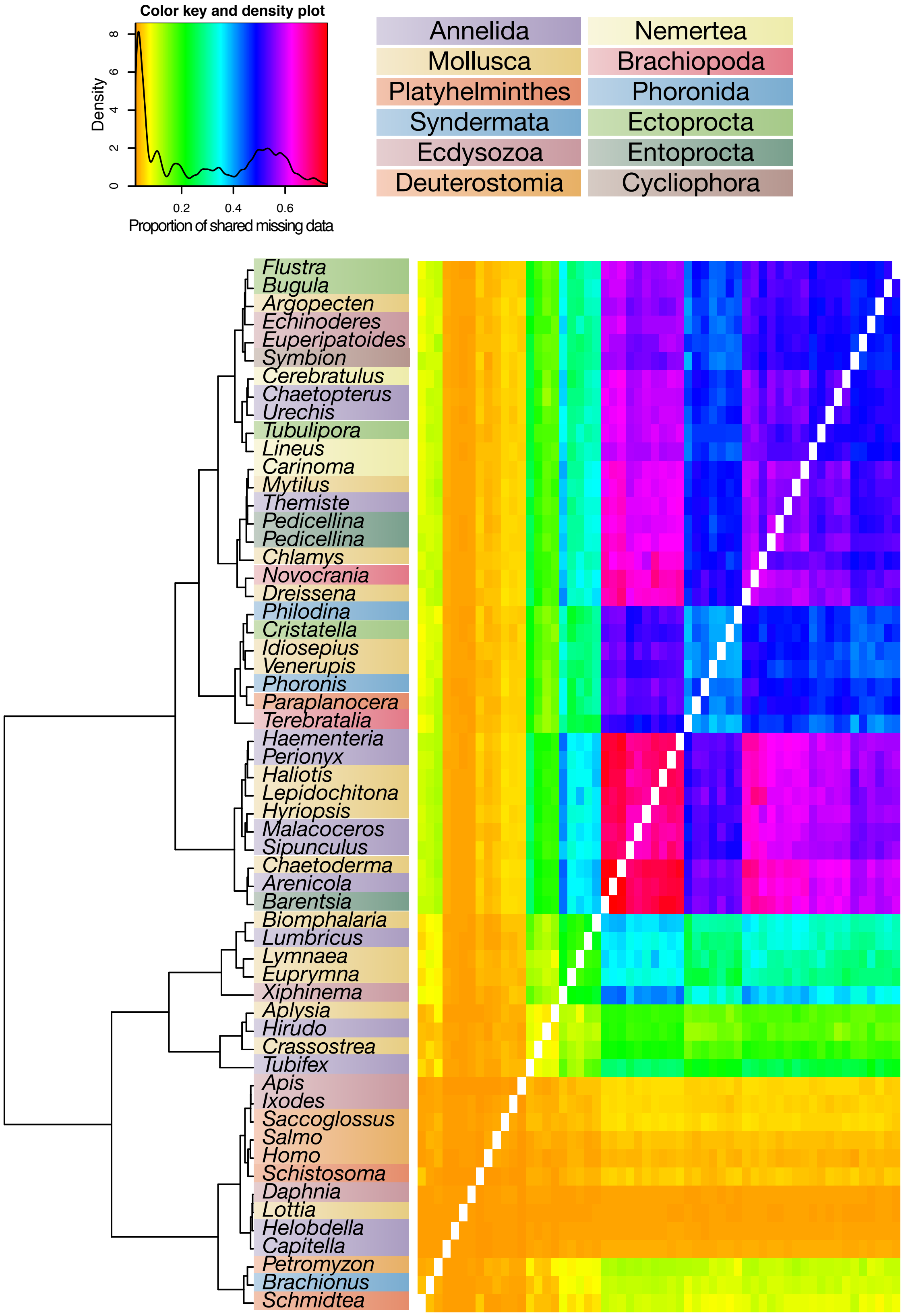

Supplementary Fig. S1. Heat map analysis combined with hierarchical clustering of the complete dataset of the degree of overlap in missing data shared between taxa. The order of the taxa from left to right along the x-axis is the same as from bottom to top along the y-axis. The higher taxonomic unit of each species is highlighted as indicated in the legend on top. Colours in the heat map indicate proportion of shared missing data ranging from 0 (orange) to 0.8 (red) (see key in upper left corner). The density distribution of the propotions is given in the upper left corner.
